# Intraoperative microscopic autofluorescence detection and characterization in brain tumors using stimulated Raman histology and two-photon fluorescence

**DOI:** 10.3389/fonc.2023.1146031

**Published:** 2023-05-10

**Authors:** Gina Fürtjes, David Reinecke, Niklas von Spreckelsen, Anna-Katharina Meißner, Daniel Rueß, Marco Timmer, Christian Freudiger, Adrian Ion-Margineanu, Florian Khalid, Konstantin Watrinet, Christian Mawrin, Andriy Chmyrov, Roland Goldbrunner, Oliver Bruns, Volker Neuschmelting

**Affiliations:** ^1^ Department of General Neurosurgery, Center of Neurosurgery, University Hospital Cologne, Cologne, Germany; ^2^ Helmholtz Zentrum München, Neuherberg, Germany; ^3^ National Center for Tumor Diseases (NCT/UCC), Dresden, Germany: German Cancer Research Center (DKFZ), Heidelberg, Germany; Medizinische Fakultät and University Hospital Carl Gustav Carus, Technische Universität Dresden, Dresden, Germany; Helmholtz-Zentrum Dresden-Rossendorf (HZDR), Dresden, Germany; ^4^ Department of Stereotaxy and Functional Neurosurgery, Center of Neurosurgery, University Hospital Cologne, Cologne, Germany; ^5^ Invenio Imaging, Inc., Santa Clara, CA, United States; ^6^ Medical Faculty, University Heidelberg, Heidelberg, Germany; ^7^ University Hospital Magdeburg, Institute of Neuropathology, Magdeburg, Germany

**Keywords:** brain tumor, autofluorescence, artificial intelligence, stimulated Raman histology, fluorescence-guided surgery (FGS)

## Abstract

**Introduction:**

The intrinsic autofluorescence of biological tissues interferes with the detection of fluorophores administered for fluorescence guidance, an emerging auxiliary technique in oncological surgery. Yet, autofluorescence of the human brain and its neoplasia is sparsely examined. This study aims to assess autofluorescence of the brain and its neoplasia on a microscopic level by stimulated Raman histology (SRH) combined with two-photon fluorescence.

**Methods:**

With this experimentally established label-free microscopy technique unprocessed tissue can be imaged and analyzed within minutes and the process is easily incorporated in the surgical workflow. In a prospective observational study, we analyzed 397 SRH and corresponding autofluorescence images of 162 samples from 81 consecutive patients that underwent brain tumor surgery. Small tissue samples were squashed on a slide for imaging. SRH and fluorescence images were acquired with a dual wavelength laser (790 nm and 1020 nm) for excitation. In these images tumor and non-tumor regions were identified by a convolutional neural network that reliably differentiates between tumor, healthy brain tissue and low quality SRH images. The identified areas were used to define regions.of- interests (ROIs) and the mean fluorescence intensity was measured.

**Results:**

In healthy brain tissue, we found an increased mean autofluorescence signal in the gray (11.86, *SD* 2.61, *n*=29) compared to the white matter (5.99, *SD* 5.14, *n*=11, *p*<0.01) and in the cerebrum (11.83, *SD* 3.29, *n*=33) versus the cerebellum (2.82, *SD* 0.93, *n*=7, *p*<0.001), respectively. The signal of carcinoma metastases, meningiomas, gliomas and pituitary adenomas was significantly lower (each *p*<0.05) compared to the autofluorescence in the cerebrum and dura, and significantly higher (each *p*<0.05) compared to the cerebellum. Melanoma metastases were found to have a higher fluorescent signal (*p*<0.01) compared to cerebrum and cerebellum.

**Discussion:**

In conclusion we found that autofluorescence in the brain varies depending on the tissue type and localization and differs significantly among various brain tumors. This needs to be considered for interpreting photon signal during fluorescence-guided brain tumor surgery.

## Introduction

Fluorescent labeling of tumor cells for guiding tumor removal is an emerging technique applied in various oncological surgeries. The visual contrast enabling surgical guidance is determined by the signal-to-noise ratio, the relation of light emission by the fluorescent label itself, and the background fluorescence, the autofluorescence of the tissue. A high level of tissue autofluorescence may reduce sensitivity and specificity of the fluorescent probe. In addition, the varying extent of autofluorescence interferes with the signal interpretation (1–3). Autofluorescence describes the natural emission of light by biological tissues subsequent to the absorbance of light. All tissues have fluorescent components that differ in their optical properties due to their chemical structures.

Most of the endogenous fluorophores such as flavin and/or pyridine nucleotides are involved in cellular energy metabolism or lipopigments usually originate as by-products of lipid oxidation (4, 5). The extracellular matrix substantially contributes to autofluorescence due to the relatively high quantum yield of collagen and elastin. By monitoring biochemical processes, the autofluorescence itself already provides diagnostic information in multiple pathologies (e.g., retinal degeneration or atherosclerosis) (6–9). The potential different metabolism in tumor cells and healthy stroma may lead to different accumulation of endogenous fluorophores that cause differences in autofluorescence. Autofluorescence in tumors shows a wide range from increased autofluorescence signal [e.g., melanoma compared with skin (10, 11)] to reduced signal [e.g., gastrointestinal malignancies or soft tissue carcinomas (12–14)] in the respective organs of origin. Data regarding the autofluorescence of the brain and its neoplasia are scarce (15) despite the use of 5-aminolevulinic acid (5-ALA) for fluorescence-guided high-grade glioma surgery as the current state of the art.

Further knowledge of the autofluorescence phenomena of the brain and its tumors is needed to better understand the influence of potential endogenous fluorophores in fluorescence-guided surgery (FGS). This study aims to assemble and characterize autofluorescence of the brain and its various neoplasia by using an intraoperative label-free fiber-laser-based microscope to enable stimulated Raman histology (SRH) (16–23) combined with two-photon fluorescence. By combining the two well-known optical events of Raman scattering and excitation by absorption of light, this workflow creates virtual hematoxylin-and-eosin-stained-like images with corresponding fluorescence images out of unprocessed tissue specimen within a few minutes during surgery. Using this technique, we provide a descriptive and semiquantitative analysis of autofluorescence patterns on a microscopic level that may interfere with the signal from exogenous fluorophores applied in neurooncological surgery.

## Materials and methods

In a prospective single-center observational study design, patients were recruited in 2021 after an interdisciplinary neuro-oncological tumor board indicated resective surgery or stereotactic biopsy as approved by the local ethics committee (Nr. 21-1238). All patients gave their written informed consent for the scientific use of their data according to the European law.

Inclusion criteria were as follows: [1] suspected tumorous lesions of the central nervous system, [2] age over 18 years, and [3] the patient is willing and able to give informed consent for participation. Exclusion criteria were as follows: [1] Patient does not agree to participate in the study or [2] is unable to or unwilling to give informed consent. Specimen samples were excluded if the collected specimen was inadequate, for example, broken slide or specimen sample size under 1.7-mm diameter for SRH imaging. Tumor samples from patients who preoperatively received 5-ALA were excluded as well. All specimen samples obtained were imaged timely after collection by the intraoperative label-free fiber-laser-based stimulated Raman scattering (SRS) microscope and evaluated by the convolutional neural network (CNN) (18) as the output class [1] tumor, [2] non-tumor, or [3] low quality. A senior board-certified neuropathologist experienced in analyzing SRH images reviewed all samples, confirmed the non-tumor tissue as healthy tissue, and identified white and gray matter regions in the SRH images.

### Specimen collection, intraoperative stimulated Raman histology and fluorescence imaging method

**Figure 1:**
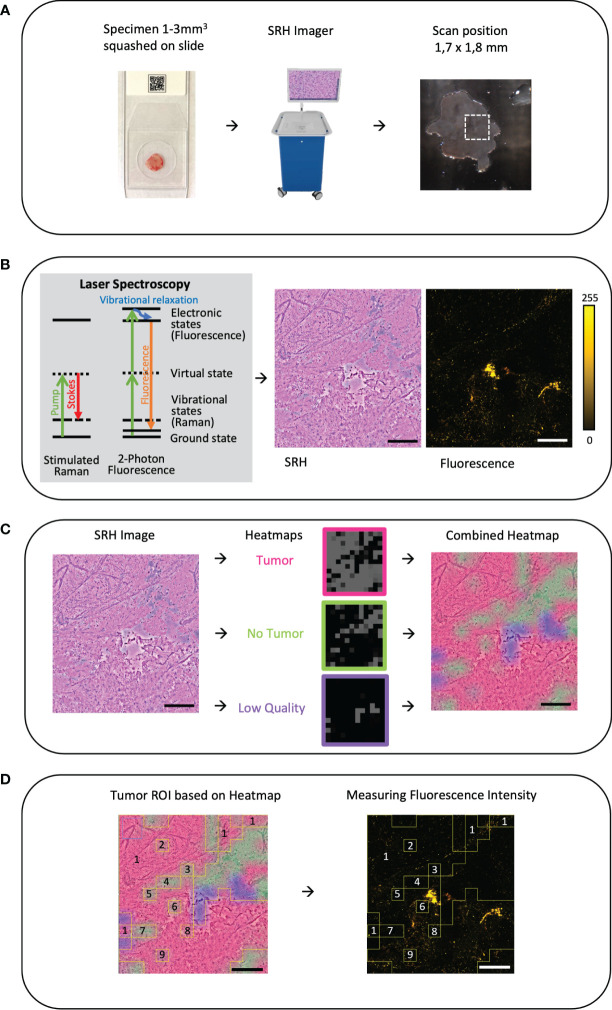
**(A)** Workflow and sample preparation. Small tumor sample is squashed onto a slide under a coverslip and then it is placed in the fiber-laser based SRS microscope (Invenio Imaging Inc, Santa Clara, CA, USA). On a picture of the sample on the slide the field of view (FOV 1.7 × 1.8mm) and position can be chosen. Here, on average, three different areas were randomly picked. **(B)** By combing two different optical events: [1] Raman scattering that leads to vibrational state changes and wavelength shifts and [2] excitation by absorption of light leading to fluorescence emission, virtual hematoxylin-and-eosin-stained-like images with corresponding fluorescence images are generated. **(C)** Based on an established convolutional neuronal network (CNN) differentiation of tumor (red), non-tumor (green), and low-quality (blue) SRH image is performed and the heatmap is created as an overlay on the SRH image (18). **(D)** Using the CNN heatmap region of interests (ROIs) were created and overlayed onto the corresponding autofluorescence image to determine the mean fluorescence intensity in the corresponding ROI. In this example, the ROIs 2–9 are subtracted from the ROI 1 to measure the mean tumor intensity. Large image scale bars = 450 µm; count scale: 0–255 counts.

**Figure 2:**
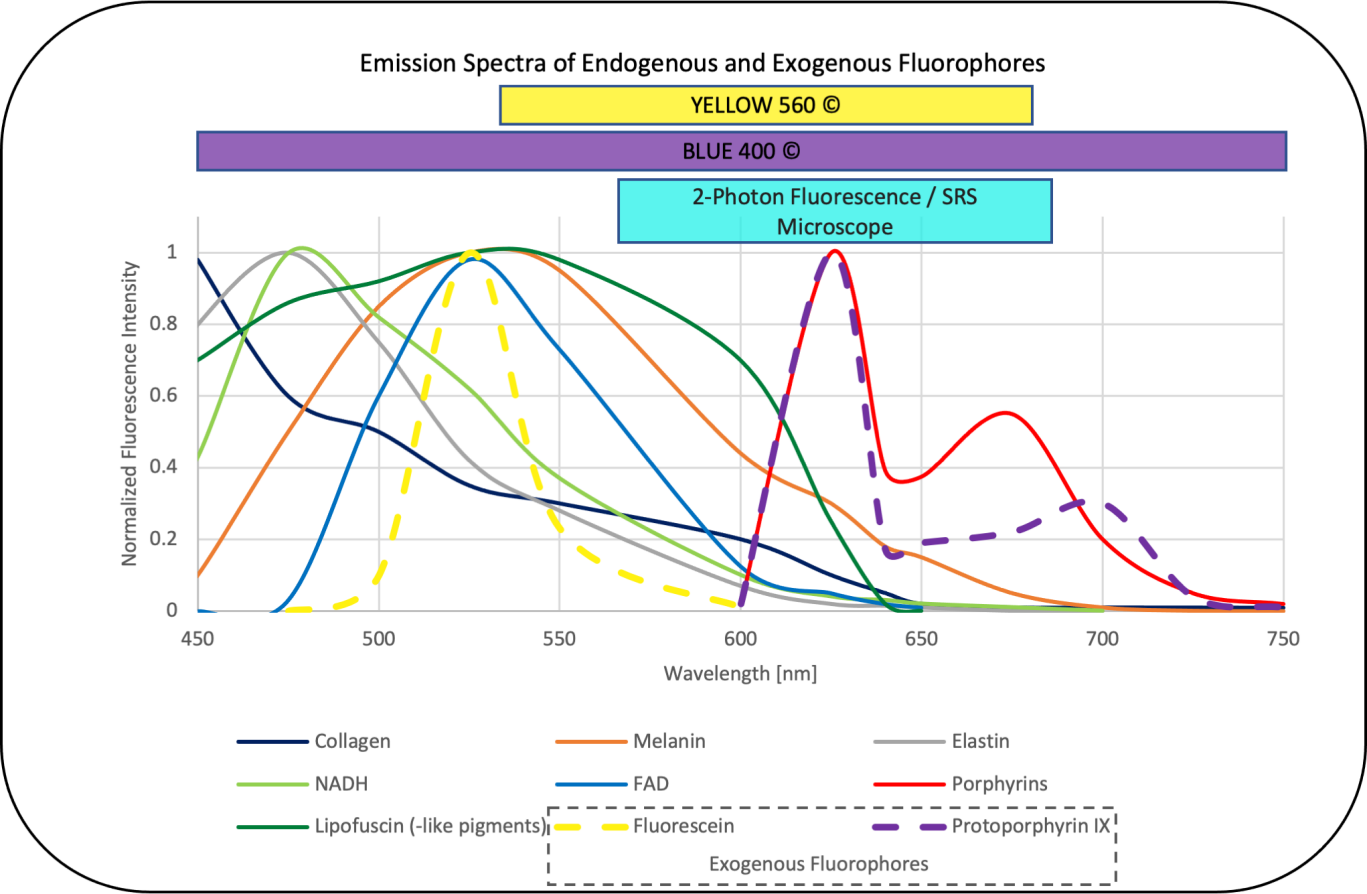
Emission spectra of endogenous fluorophores [collagen, melanin, elastin, NADH, FAD, lipofuscin (-like pigments), porphyrins] (26–32) that may interfere with the overlapping emission of the exogenous fluorophores fluorescein and PPIX (33) commonly applied in neurooncological surgery. The corresponding range focused filter setup designed in the dual modal SRS and two-photon fluorescence microscope applied in this study also overlaps with the emission detection filters commonly applied in FGS, (here, YELLOW560© and BLUE400©, Carl Zeiss Meditec, Oberkochem, Germany) (34, 35) the values are normalized to the maximum intensity in the range between 450 and 750 nm and emission was observed with one-photon excitation between 330 and 405 nm for the endogenous fluorophores as well as PPIX and 480 nm for fluorescein.

Tissue specimens were intraoperatively sampled from the tumor lesion itself or from the approach area in open surgery. For virtual imaging of fresh specimens, a small unprocessed specimen sample (1–3 mm³ in size) was squashed onto a glass slide with the help of the coverglass and imaged by a fiber-laser-based SRS microscope (Invenio Imaging Inc, Santa Clara, CA, USA) (**Figure 1A**). The microscope consists of four main components: [1] a fiber-coupled microscope, [2] a dual-wavelength (790 and 1020 nm) fiber-laser module, [3] a laser and microscope control module, and [4] a computer for data displaying, processing, and application of a convolutional neuronal network. Depending on the composition of proteins and lipids, the tissues show differences in the Raman scattering spectrums that lead to the contrast in the images. Here, two Raman shift wave numbers, 2845 cm^−1^ (CH2/lipid channel) and 2940 cm^−1^ (CH3/protein and ribonucleic acids) were used for image acquisition. The third channel combines the two Raman shift wave numbers to produce an SRH image. By acquiring a mosaic out of 400 × 400 μm SRH field of views at a speed of 2 s per frame and subsequent image calculation, a virtual H&E like image in the size of 3.06 mm^2^ was created (17–25). The two-photon fluorescence signal is detected in reflection of the sample (**Figure 1B**). During the pre-processing for the CNN algorithm, each pixel is divided by the average brightness of the entire patch to calibrate the intensity. As such, absolute intensity is removed as a variable and the algorithm functions based on relative changes within the patch. The power in focus is approximately 120 and 160 mW for the 790 and 1020 nm beam, respectively. The emission is separated from the dual-wavelength excitation beams using a 700-nm long-pass dichroic mirror (Edmund Optics 69-903), filtered with a high optical density (OD) short-pass filter (Thorlabs FESH0700), and detected with a dual-channel photo-multiplier tube (PMT) detector (Hamamatsu H10723-20). The detection channels are band-pass filtered with a 590-nm filter (570–613 nm, Edmund Optics 67-020) and 640-nm filter (603–678 nm, Edmund Optics 67-022) and displayed in pseudo-color in the green and red image channel of the resulting RGB (red, green, and blue color model) image, respectively. The emission detection range of 570 nm up to 678 nm was chosen in our setup, allowing to detect potential endogenous contributors in the common emission range of protoporphyrin IX (PPIX) and fluorescein applied in clinical FGS routine (**Figure 2**). For fluorescence detection, both beams of the dual-wavelength laser systems (790 and 1020 nm) were enabled but the time-delay was detuned by ~10 ps to avoid generation of coherent anti-Stokes Raman scattering (CARS) signal, which would be detectable in the 640-nm channel and can overwhelm the autofluorescence signal.

### Generating tumor heatmaps by a convolutional neural network

To distinguish between tumor, non-tumor, and low-quality SRH image, an already established and validated CNN was applied as previously described in detail (18). In brief, the CNN was trained on an annotated dataset of 570 whole-slide SRH images, representing the full spectrum of neurooncological surgery. The training was stopped after the preliminary validation accuracy exceeded 95% and the loss was lower than 0.10. The CNN was re-trained a second and a third time with different random seeds on the same external dataset for reliability and ROC analysis. The final probability heatmap of the presence of tumor (red), non-tumor (green), and low-quality (blue) is coded on top of the SRH image as a semi-transparent overlay (**Figure 1C**). Low-quality regions in SRH images were defined as areas that cannot be identified as tumor or non-tumor by the CNN. This can be caused, for example, by detritus, tissue disruptions due to squeezing, scan artifacts, photon absorbance of blood, and even fluid areas without any tissue. Areas that had low quality as first class in the CNN were excluded from the analysis of the final whole-slide prediction. As such, only “high quality” area probabilities were averaged over the whole image for final prediction. A quality control of the tumor heatmap was done by the observers.

### Creating region of interests to measure fluorescence

Outlined by the tumor heatmap, we were able to distinguish between tumor, non-tumor, and low-quality SRH Image. Image postprocessing ROI was created based on the heatmap (ImageJ, open source) and then transferred to the corresponding fluorescence image to measure the mean fluorescence intensity per ROI in counts (**Figure 1D**).

### Statistical analysis

Statistical analysis was performed using OriginLab (Northampton, Massachusetts, USA). Continuous values are stated in mean and with standard deviation for descriptive statistics. For *p*-value calculation, the Mann-Whitney *U* test was used for not normally distributed data. *P*-values below 0.05 were considered statistically significant.

## Results

### Demographics and baseline characteristics

We were able to include 81 patients who underwent brain tumor surgery in 07-08/2021 at our center; 37 patients (45.6%) were male. Sixty-four patients underwent open surgery (79%), whereas 17 patients were stereotactically biopsied (21%).

The final histopathological diagnoses were various carcinoma and melanoma metastases in 26 patients (32%), benign to atypic meningioma in 13 patients (16%), glial tumor in 15 patients (18.5%), pituitary adenoma including apoplectiform ones in six (7.4%), lymphoma in six (7.4%), and craniopharyngioma in two (2.4%) patients. Other mixed entities were, for example, Rathke cleft cyst, hemangioblastoma, lipoma, and epidermoid cyst in eight patients (9.8%). Tumors from five patients (6.1%) were excluded due to 5-ALA application, but the normal brain samples of the tumor approach were included for analysis (**Table 1**).

**Table 1:** Numbers of patients and images are listed for all examined tumor entities.

Tumor Entity	Patients	images
Meningioma	13	64
CNS WHO 1	11	56
Atypical CNS WHO 2	2	8
Brain Metastasis	26	151
Colorectal Adenocarcinoma	1	6
Gastroesophageal Adenocarcinoma	1	4
Cervix Carcinoma	2	15
Kidney Cell Carcinoma	1	3
Mammary Carcinoma	1	5
Multiple Myeloma	1	6
Pulmonary Adenocarcinoma	7	49
Large Cell Neuroendocrine Tumor	1	6
Small Cell Lung Carcinoma	1	3
Squamous Epithelial Carcinoma	2	15
Salivary Duct Carcinoma	1	4
Sarcoma	1	4
Melanoma	3	11
Dedifferentiated Cancer of unknown primary (CUP)	3	20
Glial tumors	15	47
IDH mutant Astrocytoma CNS WHO 2-4	2	5
Glioblastoma	4	11
Oligodendroglioma CNS WHO 3	1	2
Gliosis and Glioma Infiltration	2	2
Pilocytic Astrocytoma CNS WHO 1	2	3
Ependymoma CNS WHO 2	2	14
Medulloblastoma	2	10
Pituitary adenoma	6	30
Apoplectiform	1	6
Rest	5	24
Nerve Fiber Tumors	2	11
Schwannoma	1	6
Ganglioneurinoma Craniopharyngeoma	1	5
Craniopharyngeoma	2	9
Papillary	1	6
Adamantinomatous	1	3
Others		
Epidermoid cyst	1	4
Lymphoma	6	14
Hemangioblastoma	1	8
Lipom	1	2
Mixed Tumor Skin	1	4
Multiple Sclerosis Rathke cleft cyst	1 1	1 2
Normal Brain	5	13

In total, 162 specimens were collected and 397 SRH and consecutive fluorescence images were acquired using the intraoperative laser imaging system. Specimens have been scanned in multiple random areas, in an average three areas per slide. Forty fluorescence images of normal brain tissue derived from 18 patients were included in our study, which were collected from the surgical access route to malignant intraaxial tumors.

### Evaluation of the fluorescence data

**Figure 3:**
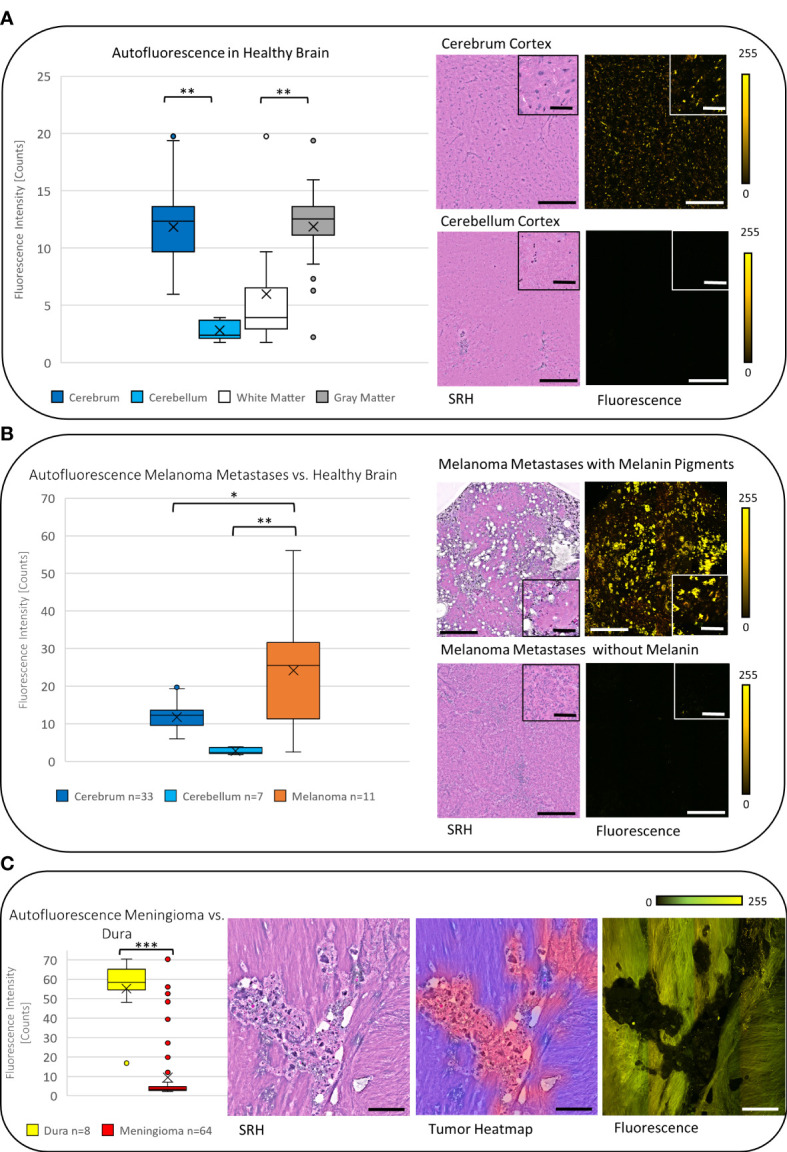
**(A)** The boxplot diagram shows the significantly increased mean autofluorescence intensity in the cerebrum and the cerebellum and in the gray matter compared with the white matter (*p* < 0.001), respectively. The SRH images with the corresponding fluorescence images for the cerebrum and the cerebellum demonstrate the measured difference. **(B)** On average the autofluorescence intensity in melanoma metastases was found to be higher than the autofluorescence intensity in cerebrum and cerebellum (*p* < 0.01). **(C)** Meningiomas show a low autofluorescence signal, that leads to significant negative contrast in the fluorescence image compared with the non-tumorous dura yielding a high autofluorescence signal (*p* < 0.001). The tumor heatmap shown here serves as an illustration of the control for the tumor margins by the CNN. Large image scale bars = 450 µm; inset image scale bars = 100 µm; count scale: 0–255 counts. * = p ≤ 0.05, ** = p ≤ 0.01, *** = p ≤ 0.001.

Focusing on the non-tumorous brain tissue, a significantly higher mean autofluorescence signal was noticed in the cerebrum with 11.83 counts (*SD* = 3.29, *n* = 33) compared with the cerebellum with 2.82 counts (*SD* = 0.93, *n* = 7, *p* < 0.001). Most of the autofluorescence signal was detected in the neuronal bodies especially compared with the axons; therefore, the mean autofluorescence signal was significantly higher in the gray matter (mean = 11.86, *SD* = 2.61, *n* = 29) compared with the white matter (mean = 5.99, SD = 5.14, *n* = 11, *p* < 0.01) (**Figure 3A**).

**Figure 4:**
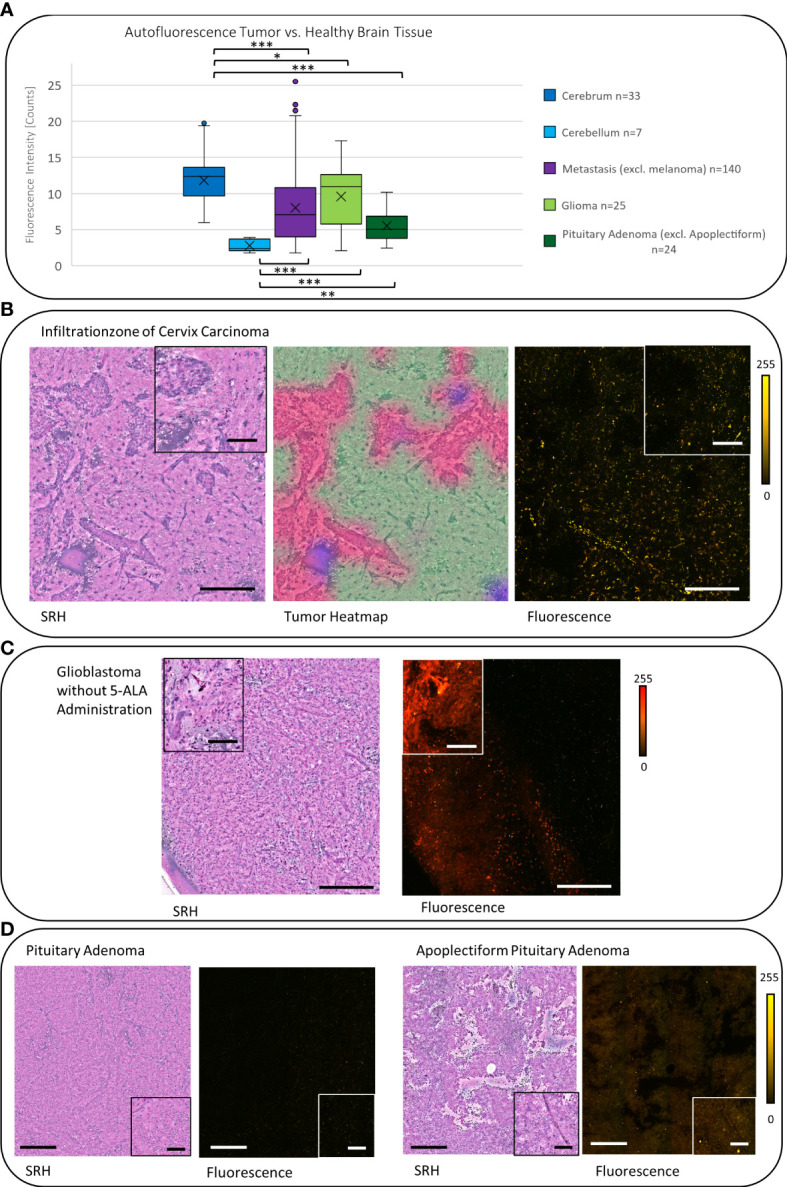
**(A)** The signal of carcinoma metastases, gliomas, and pituitary adenomas were significantly lower (each *p* < 0.05) compared with the autofluorescence in the cerebrum. Whereas they showed a significantly higher signal (except of meningiomas) (each *p* < 0.05, gliomas *p* < 0.001) compared with the cerebellum. **(B)** The SRH image shows the tumor infiltration zone of a cervix carcinoma. The tumor areas can be controlled in the tumor heatmap. In the fluorescence image, the resulting negative contrast of the tumor to the healthy brain is visible. **(C)** Glioblastoma sample from a stereotactic biopsy (without administration of 5-ALA) shows a strong reddish autofluorescence signal. **(D)** Pituitary adenomas in general showed a low autofluorescence intensity (compare 4A), but in apoplectiform pituitary adenomas, we observed an increased autofluorescence intensity. Large image scale bars = 450 µm; inset image scale bars = 100 µm; count scale: 0–255 counts. * = p ≤ 0.05, ** = p ≤ 0.01, *** = p ≤ 0.001.

In carcinoma metastases (excluding melanoma metastases), the mean autofluorescence intensity was 7.58 counts (*SD* = 4.75, *n* = 140) and was significantly lower compared with the signal in the cerebrum and higher compared with the cerebellum (both *p* < 0.001). This leads to a negative contrast between the tumor tissue and the healthy tissue in the tumor margins within the cerebrum (**Figures 4A, B**). The melanoma metastases showed a mean autofluorescence intensity of 24.21 counts (*SD* = 16.88, *n* = 11) and were significantly brighter in comparison with both the cerebrum (*p* < 0.05) and the cerebellum (*p* < 0.01). Striking fluorescence was observed from the melanin pigments in yellow that were produced by some melanomas, whereas low or not pigmented melanomas presented autofluorescence comparable with carcinoma metastases (**Figure 3B**).

In gliomas, we observed a mean autofluorescence of 10.08 counts (*SD* = 4.16, *n* = 25) that was still less fluorescent than the signal in the cerebrum (*p* < 0.05) but again more fluorescent than the autofluorescence of the cerebellum (*p* < 0.001). Regarding the malignancy, it seems like glioblastoma show a higher mean autofluorescence (mean = 11.4 counts, *SD* = 3.58, *n* = 11) compared with anaplastic IDH mutant astrocytomas (mean = 5.82 counts, *SD* = 2.4, *n* = 5) or oligodendrogliomas (mean = 5.06 counts, *SD* = 0.14, *n* = 2) in our small cohort (*p* < 0.01). Interestingly, depending on the localization, some label-free glioma cases derived from stereotactic biopsy and, thus, not receiving 5-ALA prior to surgery presented a strong red autofluorescence (**Figure 4C**).

Due to the different histopathological and stromal features, meningiomas showed a broad range of autofluorescence intensity. On average, the autofluorescence was 9.42 counts (*SD* = 15.27, *n* = 64) and therefore significantly less fluorescent than the cerebrum (*p* < 0.001). We observed psammoma bodies and fibrous material causing a high autofluorescence signal, whereas most of the meningiomas showed low autofluorescence (see **Supplemental Figure S1**). From the surgical guidance point of view, the autofluorescent contrast between meningioma and the dura is more valuable than to the surrounding brain tissue, at least in most meningioma resection cases. The dura itself shows a homogenous and high autofluorescence signal in mean 55.22 counts (*SD* = 17.07, *n* = 8) and is significantly more fluorescent than meningiomas (*p* < 0.001), leading to a negative contrast of the tumor to the non-tumorous dura (**Figure 3C**).

Non-apoplectiform pituitary adenomas show a mean autofluorescence signal of 5.52 counts (*SD* = 4.46, *n* = 24) that is significantly lower than the mean autofluorescence signal to the cerebrum (*p* < 0.001) and significantly higher compared with the cerebellum (*p* < 0.01). Probably due the hemorrhages in apoplectiform pituitary adenomas, there is an increase in the mean autofluorescence signal up to 15.03 counts (*SD* = 2.49, *n* = 6) (**Figure 4D**).

Focusing on the differences in autofluorescence levels across single specimen, we observed a mean standard deviation of 1.29 counts per specimen (median = 0.62 counts, range: 0.01–10 counts, *n* = 148) with an average of 2.7 scans per specimen. The intra-patient variability was relatively low with a mean standard deviation of 1.72 counts per patient (median = 0.85 counts, range: 0.1–11.75 counts; *n* = 84) with an average of 4.58 scans per patient taking the sampling (healthy brain, infiltration zone, and tumor) into account. In healthy brain, the mean standard deviation with 0.7 counts (median = 0.34 counts, range: 0.25–1.75 counts, *n* = 10) was even lower.

## Discussion

This study provides a new understanding of the autofluorescence of the brain and its tumors on a microscopic level in the range of 570–678 nm, the spectral range that is most often applied for fluorescence guidance in neurooncological surgery. Autofluorescence can lead to a reduced signal-to-background ratio and impede the detection of fluorophores in fluorescent-guided surgery in the region of the tumor margins in particular.

### Endogenous fluorophores and the impact on fluorescence-guided surgery

We observed the autofluorescence of the non-tumorous brain to vary depending on the localization and showing a significant difference between the cerebellum and the cerebrum. The higher degree of autofluorescence that was present in the neuronal bodies may explain the higher autofluorescence in the gray matter compared with the neuronal axons in the white matter. If there is a physiological difference in the autofluorescence in the various lobes and basal ganglia, this cannot be answered by our limited cohort and needs to be further investigated.

Endogen fluorophores emission spectra that may interfere with the exogenous fluorophores in neurooncological surgery such as fluorescein and PPIX are summarized in **Figure 2** from the literature (26–32) and put in relation to the filters applied in clinical routine and our imaging setup, which show a clear overlap. Of note, emission spectra depicted here are based on one-photon excitation between 330 nm up to 405 nm and 480 nm for fluorescein, respectively. As we applied two-photon fluorescence with 790 and 1020 nm, we expect comparable emission spectra for the intrinsic and extrinsic fluorophores (PPIX and Fluorescein) in our settings. However, potentially altered emission spectra of endogenous fluorophores excited by one-photon 510 nm and two-photon 1020 nm excitation remains subject to further studies. As the emission range of FGS is wider than the range in our setup, an even higher impact of autofluorescence needs to be considered. A translation of the exact same currently available intraoperative fluorescence settings, in which white light is often used for excitation, is not reasonable on a microscopic level. The excitation spectrum is broad, and the necessary excitation power could lead to tissue burn of small samples. Our SRH and two-photon fluorescence microscope allows to mimic the FGS settings and track down the fluorescence on a cellular level. The descriptive and semiquantitative study on the characterization of autofluorescence phenomena in healthy brain and brain tumor tissue is limited in providing data about its direct impact on the extent and pattern of fluorescence detected in exogenous labeled tissue in FGS. Further studies comparing the extent and pattern of fluorescence in label-free and fluorophore-labeled tissues are needed.

In our cohort, most of the brain tumors showed a reduced autofluorescence signal compared with the healthy brain. These findings are in line with already published data on tumors outside and inside the central nervous system (12–14, 27, 36). An explanation regarding the reduced autofluorescence signal of malignancies is that typical endogenous fluorophores are often a product of cell aging. In tumor cells, the high rates of cell division and proliferation lead to an accumulation of relatively young cells. One example is lipofuscin or lipofuscin like lipopigments that are ascribed to physiological aging by oxidative stress and are intracellularly accumulated. Typically, they show fluorescence in the yellow to reddish spectrum, with variable intensity (**Figure 2**). The exact spectral profile depends on their heterogeneous composition including lipids, protein and retinoids, the degree of oxidation, and cross-linkage (37). It is likely that lipofuscin and other lipopigments are the origin of the autofluorescence in the gray matter of the brain (38). Moreover, the autofluorescence decrease that we observed in tumor tissues can be ascribed to the reduction of NADH amount in tumor cells due to the Warburg effect (39). By highly increased metabolization of glucose and lactate under aerobic conditions in cancerous tissue compared with healthy brain tissue the NADH concentration can be lowered. Furthermore, a decrease of FAD and an increase of the optical redox ratio (NADH/FADH) has also been observed in gliomas compared with healthy brain tissue as an indicator for the altered metabolic activity (27, 40). Some studies have shown an increased concentration of NADH and FAD, for example, in breast cancer cells (41) probably caused by mitochondrial dysfunction (42). So far, the concentration of NADH and FAD in various tumors is still a matter of debate as it is strongly connected to the current metabolism state.

We observed a high autofluorescence by fibrous tissues like the dura or stromal tumors with a higher proportion of extracellular matrix (e.g., some meningioma and some squamous cell carcinoma, or mixed skin tumors). Most likely, it is caused by elastin and collagen whose autofluorescence depends on their composition and steric organisation (**Figure 2**). For example, pyridinoline groups can be associated with collagen fibers and their degree of polymerization of monomeric chains and cross-links depend on age (43, 44). Because of its repetitive structural organization and anisotropic properties, the observed signal from structures with collagens such as the dura mater may possibly be caused by second harmonic generation (45). This is an inelastic scattering resulting in photons with twice the energy and half the wavelength of incident light (46). Due to the filter combination in our setup the detection of photons with twice the energy of the excitation laser with 790 nm and 1020 nm is not possible and by detuning the time delay sum-frequency generation cannot ensue. Therefore, the detected emission from the extracellular matrix we observed are caused by fluorescence phenomena.

A high autofluorescence was also observed in melanoma metastases, probably caused by the melanin pigments, whose fluorescence has already been examined to be in the yellow spectrum (**Figure 2**) matching our results (47). The low or not pigmented melanoma metastases presented autofluorescence is comparable to carcinoma metastases and this difference in melanomas explains the wide standard deviation we found.

Strikingly, in some malignant gliomas, we noticed varying extent of autofluorescence even without preoperative administration of 5-ALA before stereotactic biopsy. Porphyrin derivates such as PPIX, the metabolism product of 5-ALA, fluoresce in the red region at well-defined spectral positions (**Figure 2**) (48). Other than PPIX after 5-ALA administration, porphyrins involved in the heme metabolic could lead to a porphyrin associated fluorescence and a false positive signal in fluorescence-guided oncological neurosurgery (**Figure 2**). For example, in apoplectiform pituitary adenomas, we observed orange to reddish autofluorescence, possibly due to hemorrhages and an increased deposition of heme/hemoglobin. However, these are assumptions and this phenomenon needs to be further studied.

Due to the fact that we observed intense autofluorescence overlapping the spectrum of the currently available fluorophores for oncological neurosurgery (**Figure 2**), the imaging spectrum for FGS needs to be interpreted with caution and in highly autofluorescent entities, subtypes, and localizations; it should be re-considered. The near infrared (NIR) spectrum I from 700 to 1000 nm and NIR II or shortwave infrared (SWIR) from 1000 to 1700 nm in that regard is known to provide signal-to-noise advantages with less autofluorescence interference (3, 49–51). Due to less scattering, the penetration depth increases, and studies observed a substantial decrease in autofluorescence for NIR excitation and emission wavelength when compared with different ranges within the visible spectrum (3, 49, 50). The development of suitable NIR I or SWIR probes would potentially improve the signal-to-background ratio and could enable a higher sensitivity of FGS.

Our data suggest that autofluorescence may even enable label-free autofluorescence-guided surgery in the future by the endogenous fluorescence contrast between healthy and pathological tissue. These findings are in line with the results of different studies focusing on label free methods for tumor imaging that underline the potential of autofluorescence for future tumor guidance. For example, increased autofluorescence of pigmented skin and its lesions have already been successfully used for diagnosis and observation of melanomas in a small clinical cohort (11, 52). Furthermore, the negative contrast from reduced autofluorescence intensity in tumors was already observed in different intracranial tumors (27) and soft tissue sarcomas (14). Another study showed a combination of endogen (NADH) and exogen (PPIX) fluorophores to provide a new promising and sensitive tool to visualize brain tumor tissue not detectable with conventional 5-ALA (PPIX) fluorescence (53). Due to the heterogeneity of the autofluorescence signal, we also observed, in our small cohort, further studies on assessing and characterizing autofluorescence of the brain and its various tumors are needed.

### Data acquisition and analysis

The applied CNN previously showed an overall inter-rater agreement with the neuropathologist of 89.6% and an excellent internal consistency of 90.2% enabling a quick and efficient differentiation of tumor or non-tumor and non-diagnostic patches (18) well in alignment with previous studies showing excellent concordance between SRH and standard H&E-based techniques for diagnosis prediction (16, 20, 22). However, using the CNN-based tumor heatmaps as ground truth for the ROIs can lead to imperfect accuracy regarding the diagnosis of tumor or non-tumor tissue compared with the potential neuropathologists’ accuracy and has to be acknowledged as a limitation of the study.

Our data showed low intra-sample and intra-patient variability underlining the stability of the fluorescence measurements. At the same time, these data suggest that the observed differences in the fluorescence levels are likely attributed to the different tumor entities.

### Technical opportunities and limitations

Regarding technical limitations of the dual-modality setup presented here, the study was limited to provide data on specific exposure time requirements to enable sufficient contrast for the various tissue types as the exposure time was fixed in this setup. In comparison with exogenous contrast probes, the relatively low autofluorescence signal may require long exposure times that may limit its application for autofluorescence guidance in real time. Due to the simultaneous construction of two-photon fluorescence and SRH fluorescence signal may be lost by the photon split and cannot optimally be induced by the fixed light exposure times. In addition, reduced fluorescence signal as a result of bleaching effects needs to be considered due to the preceding simultaneous SRH acquisition. The color coding of the fluorescence images was performed computationally during the image processing. A detailed spectral analysis of the fluorescence signal may enable a more accurate spectral analysis of the endogen fluorophores.

Nevertheless, the advantages of the dual-modality image acquisition with a corresponding histopathological reference by SRH enabling the microscopic correlate to the fluorescence pattern in the same sample within minutes outweighs the limitations of the fluorescence detection setup. Using two-photon fluorescence microscopy perioperatively can restrict excitation to a tiny focal volume in thick samples. In that way out of focus excitation and photobleaching is reduced. Another benefit of two-photon fluorescence are the infrared lasers, which lead to less scattering in biological tissues compared with light of the visible spectrum and increase of the penetration depth (54).

In addition, the combined acquisition of autofluorescence and SRH images under controlled conditions may improve the accuracy of deep neuronal networks diagnostic predictions incorporating dual modal derived image information. This may potentially improve the accuracy in imaging-derived tissue diagnostics. Currently, sampling of tissue is still needed for the *ex situ* diagnostic imaging setup but future technical developments may enable *in situ* applications of the technique. One of the first experimental approaches in microscopic real time *in vivo* imaging is confocal endomicroscopy with visualization of tissue at high magnification without the need of tissue extraction (55–57).

## Conclusion

In conclusion, the intraoperative combination of two-photon fluorescence and SRH provides the opportunity to examine and interpret autofluorescence with high resolution on a microscopic level within a few minutes. The characterization of autofluorescence phenomena of the brain and its various tumors at the cellular level may contribute to a better understanding of the potentials and limitations of labeled fluorescence guidance in neurosurgery and may support the emerging research efforts in paving the way towards label-free autofluorescence guidance in neurooncological surgery the future.

## Data availability statement

The raw data supporting the conclusions of this article will be made available by the authors, without undue reservation.

## Ethics statement

The studies involving human participants were reviewed and approved by Ethics Committee of the Medical Faculty of the University of Cologne (Nr. 21-1238). The patients/participants provided their written informed consent to participate in this study.

## Author contributions

Conceptualization: VN, GF. Data acquisition: A-KM, NS, DRe, VN, DRu, GF. Design and evaluation of optical configuration: GF, CF, AC, OB, VN. Data analyses: GF, VN. Review of literature: GF, VN. Writing - original draft preparation: GF, VN. Writing - review, and editing: all authors. Tables and Figures: GF, VN. Supervision: VN. All authors contributed to the article and approved the submitted version.

## Supplementary material

Supplementary Figure 1 Illustration of the varying extent of autofluorescence in meningiomas **(A)** low autofluorescence in this meningothelial meningioma CNS WHO 1. **(B)** In meningothelial meningioma, a higher rate of extracellular matrix that leads to a higher autofluorescence signal comparable to the dura. **(C)** Psammoma bodies show an increased autofluorescence signal shown in this psammomatous meningioma. **(D)** This atypic meningioma shows high-autofluorescent fibers and psammoma bodies. Large image scale bars = 450 µm; count scale: 0–255 counts.

Supplementary Figure 2 Examples for autofluorescence in metastases of various primaries. **(A)** Inhomogeneous autofluorescence with pigment deposits of a squamous epithelia carcinoma metastasis. **(B, C)** Different examples of autofluorescence in pulmonary adenocarcinoma metastases. **(D)** Low autofluorescence in a sarcoma metastasis. Large image scale bars = 450 µm; count scale: 0–255 counts.

Supplementary Figure 3 Examples for autofluorescence in suprasellar tumors. **(A)** Adamantinomatous craniopharyngioma with high fluorescent keratin cysts. **(B)** Compared with that low fluorescent papillary craniopharyngioma. **(C)** Low autofluorescence of a Rathke cleft cyst. Large image scale bars = 450 µm; count scale: 0–255 counts.

Supplementary Figure 4 Illustration of autofluorescence in rare infratentorial tumors. **(A)** Reticular autofluorescence pattern in a hemangioblastoma. **(B)** Low autofluorescence in a medulloblastoma and the cerebellum without resulting contrast. **(C)** Diffuse autofluorescence of a pilocytic astrocytoma. Large image scale bars = 450 µm; count scale: 0–255 counts.

Supplementary Figure 5 Autofluorescence in different kinds of gliomas. **(A)** Spot of red autofluorescence without preoperative 5-ALA administration in an anaplastic astrocytoma CNS WHO 3. **(B)** A different example of autofluorescence in glioblastoma in the yellow to orange wavelength range. **(C)** Intermediate autofluorescence in an Oligodendroglioma CNS WHO 3. Large image scale bars = 450 µm; count scale: 0–255 counts.
