# Supplementary figures and images for: Intraoperative microscopic autofluorescence detection and characterization in brain tumors using stimulated Raman histology and two-photon fluorescence
Source: Front Oncol. 2023 May 10;13:1146031. doi: 10.3389/fonc.2023.1146031 (PMC10207900; doi:10.3389/fonc.2023.1146031)

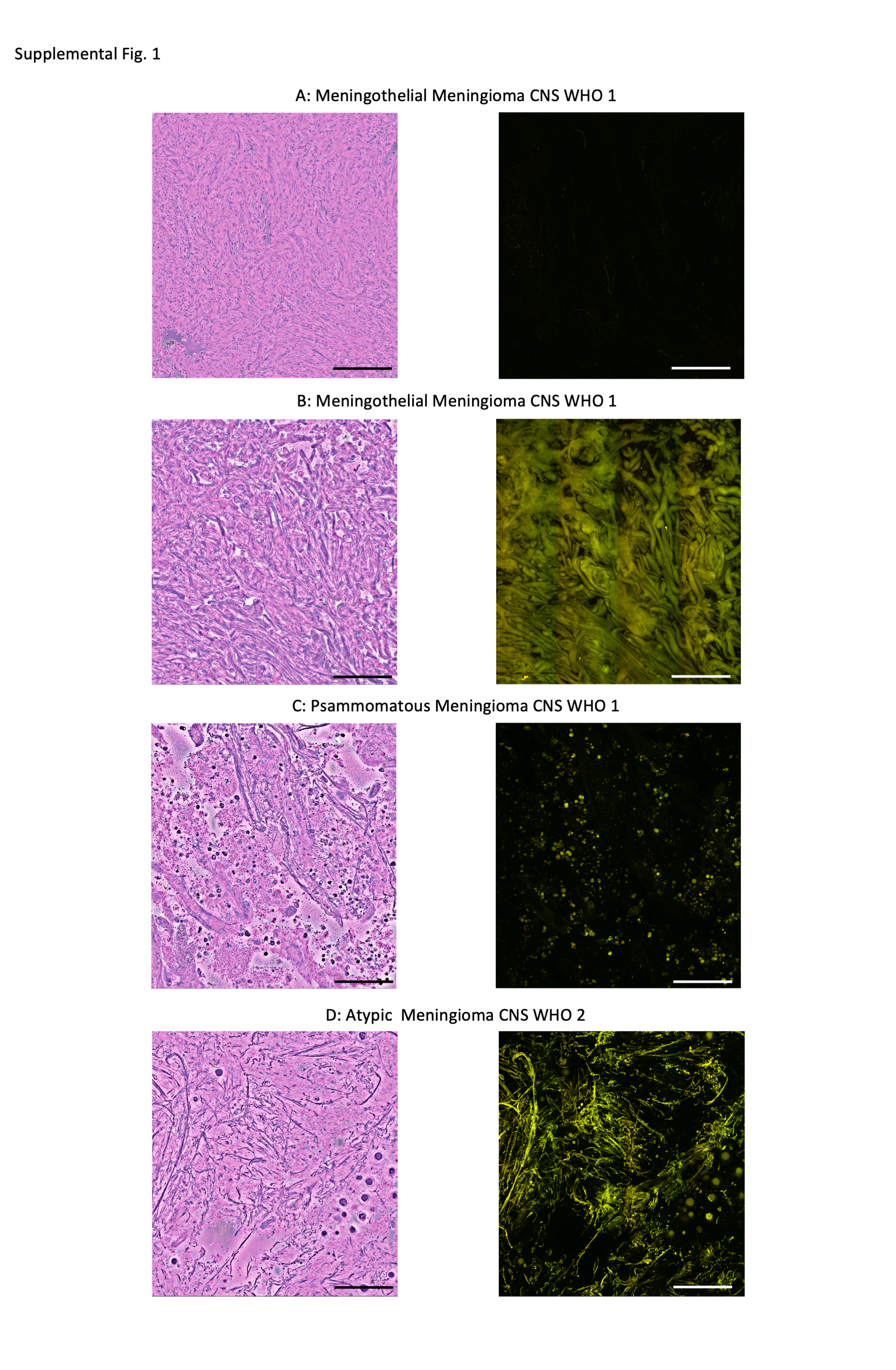

Supplement: Supplementary Figure 1 — Illustration of the varying extent of autofluorescence in meningiomas (A) low autofluorescence in this meningothelial meningioma CNS WHO 1. (B) In meningothelial meningioma, a higher rate of extracellular matrix that leads to a higher autofluorescence signal comparable to the dura. (C) Psammoma bodies show an increased autofluorescence signal shown in this psammomatous meningioma. (D) This atypic meningioma shows high-autofluorescent fibers and psammoma bodies. Large image scale bars = 450 µm; count scale: 0–255 counts. [file Image_1.tiff]

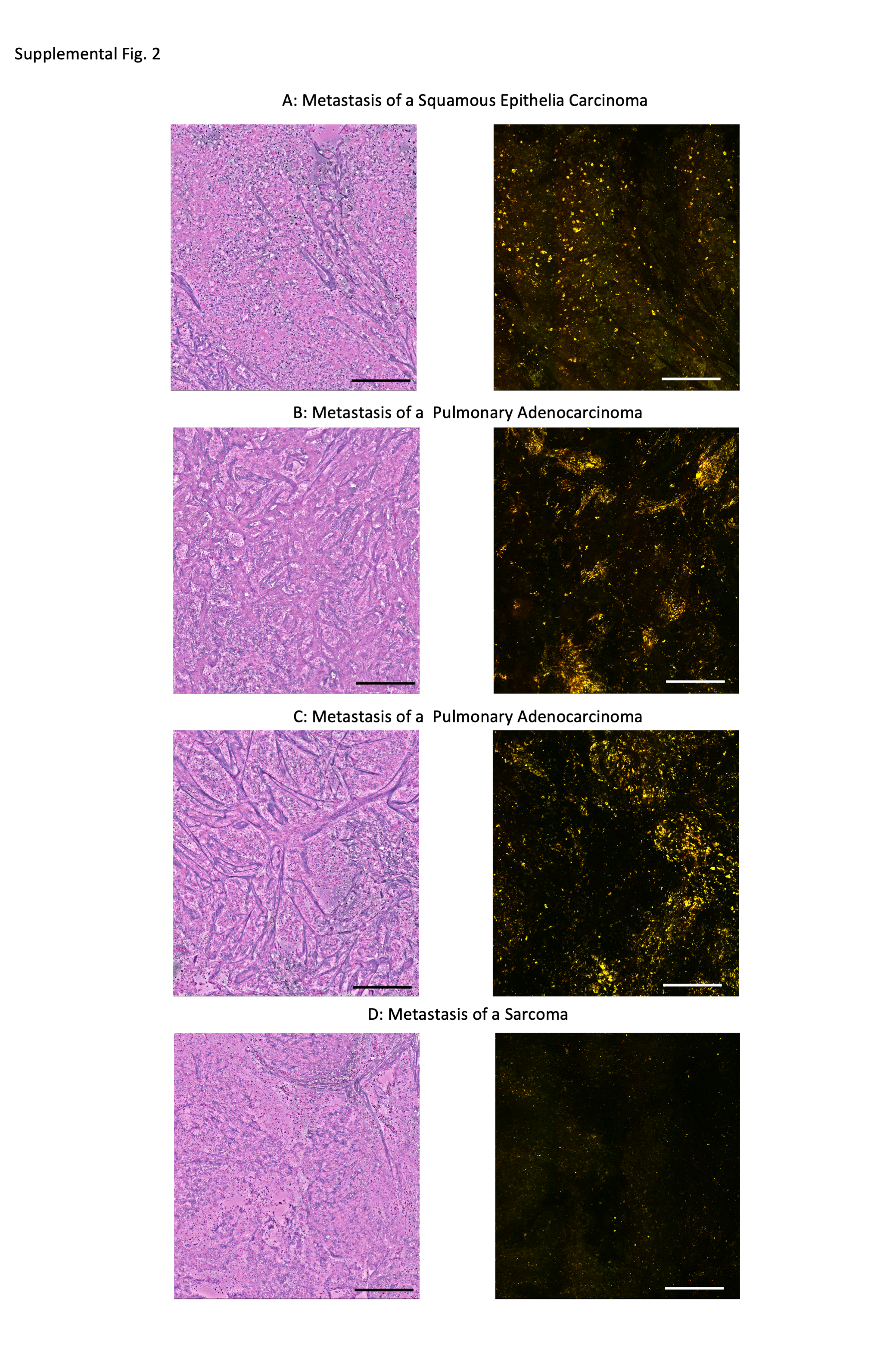

Supplement: Supplementary Figure 2 — Examples for autofluorescence in metastases of various primaries. (A) Inhomogeneous autofluorescence with pigment deposits of a squamous epithelia carcinoma metastasis. (B, C) Different examples of autofluorescence in pulmonary adenocarcinoma metastases. (D) Low autofluorescence in a sarcoma metastasis. Large image scale bars = 450 µm; count scale: 0–255 counts. [file Image_2.tiff]

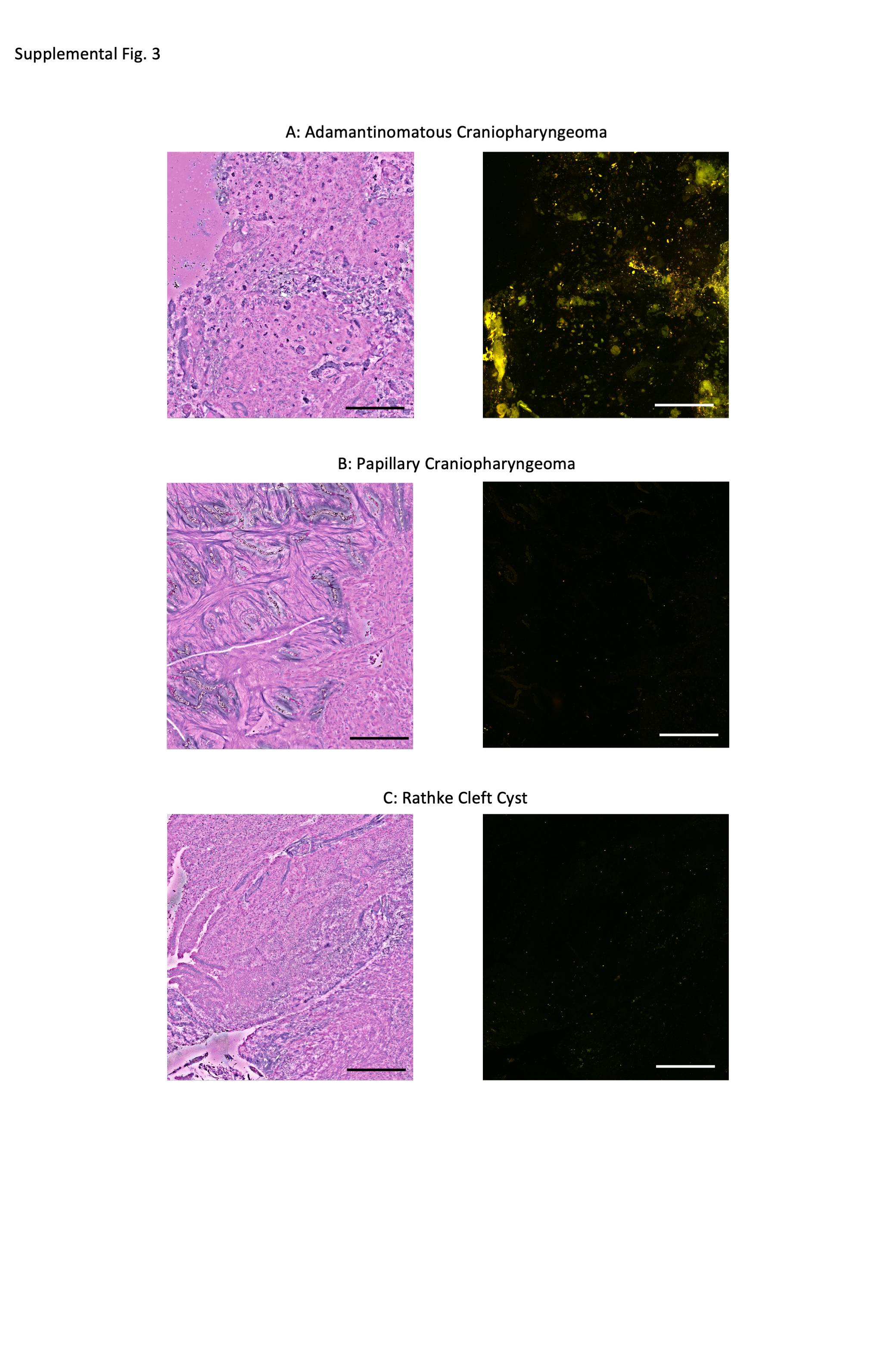

Supplement: Supplementary Figure 3 — Examples for autofluorescence in suprasellar tumors. (A) Adamantinomatous craniopharyngioma with high fluorescent keratin cysts. (B) Compared with that low fluorescent papillary craniopharyngioma. (C) Low autofluorescence of a Rathke cleft cyst. Large image scale bars = 450 µm; count scale: 0–255 counts. [file Image_3.tiff]

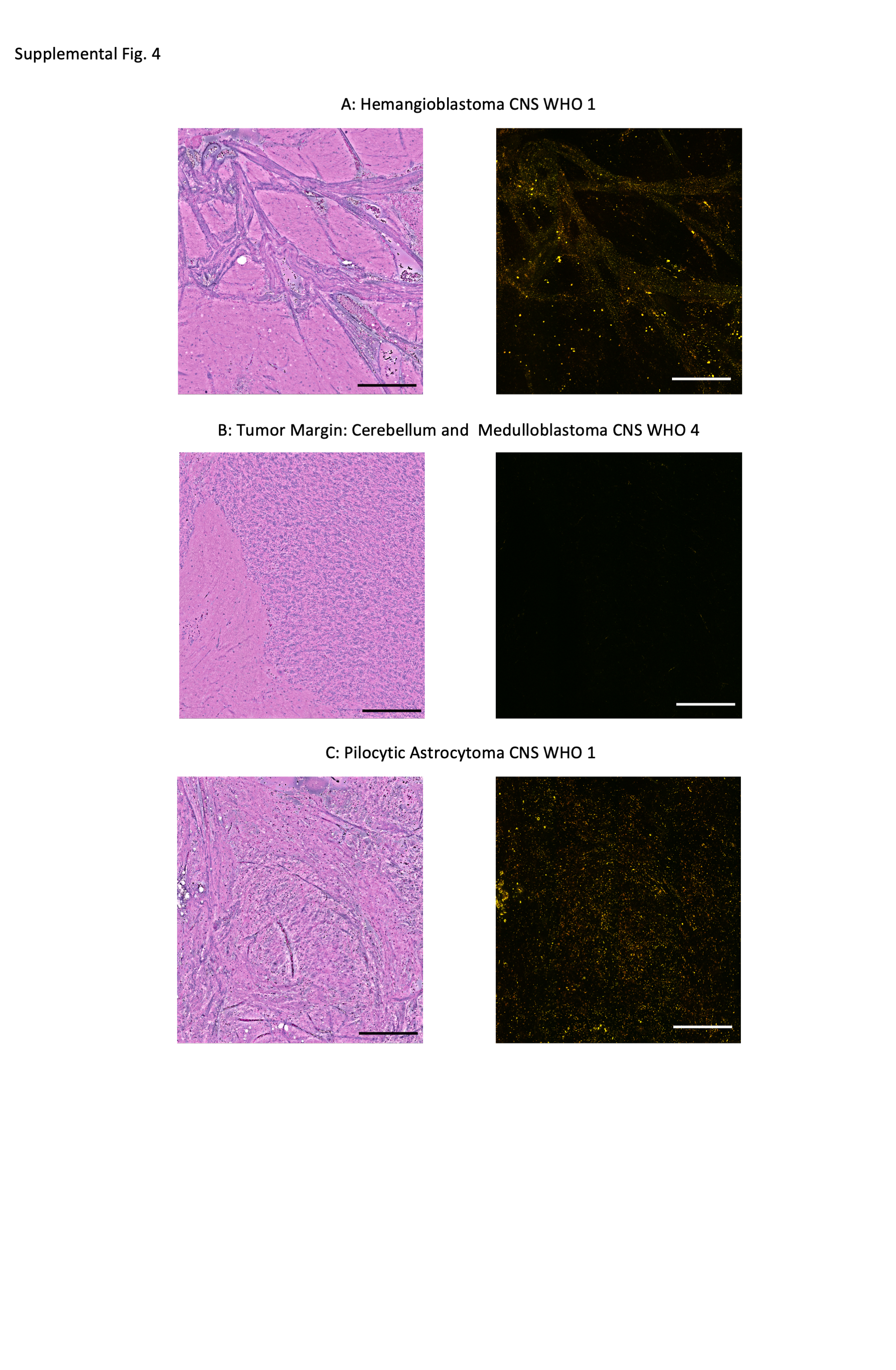

Supplement: Supplementary Figure 4 — Illustration of autofluorescence in rare infratentorial tumors. (A) Reticular autofluorescence pattern in a hemangioblastoma. (B) Low autofluorescence in a medulloblastoma and the cerebellum without resulting contrast. (C) Diffuse autofluorescence of a pilocytic astrocytoma. Large image scale bars = 450 µm; count scale: 0–255 counts. [file Image_4.tiff]

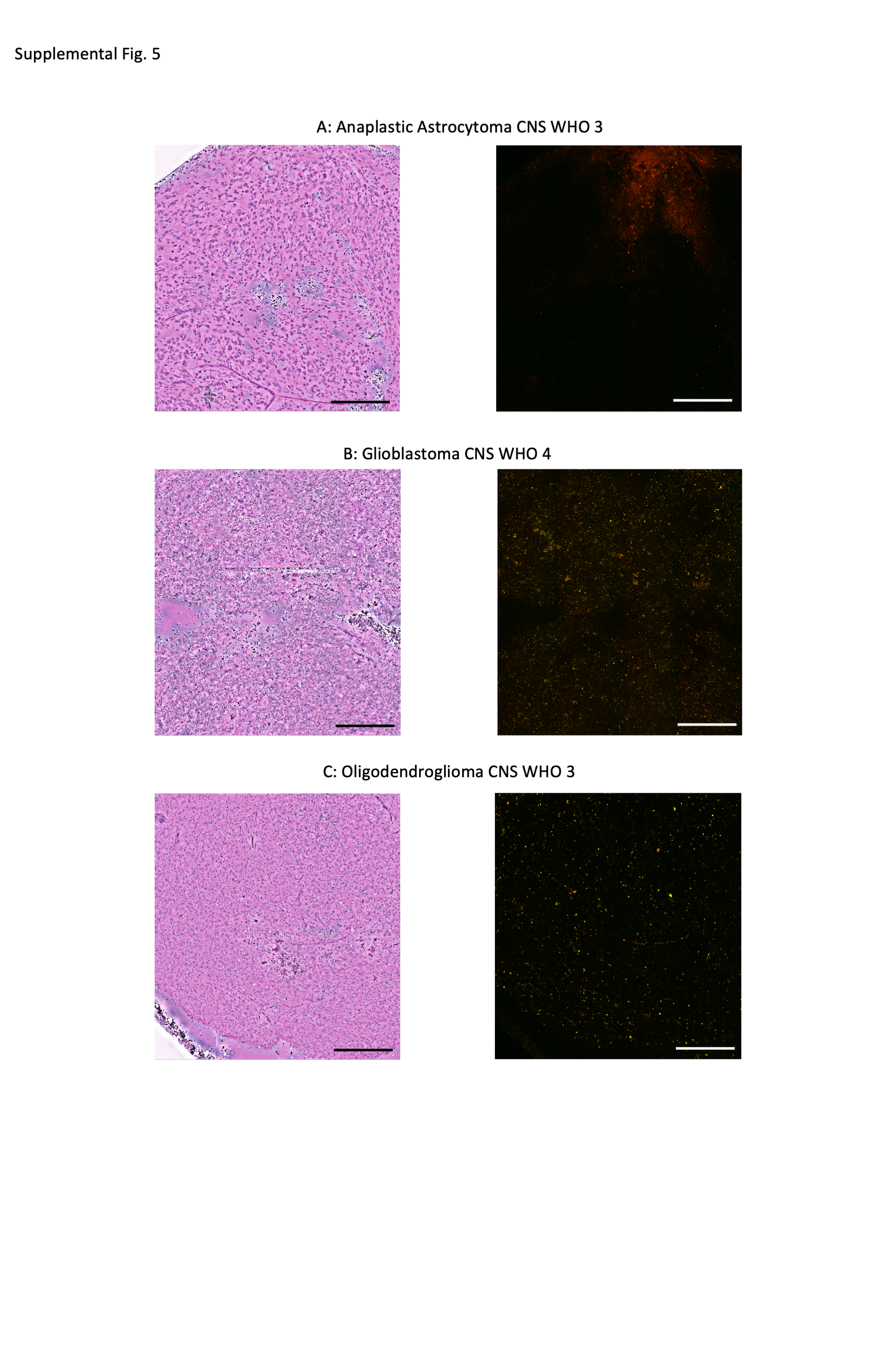

Supplement: Supplementary Figure 5 — Autofluorescence in different kinds of gliomas. (A) Spot of red autofluorescence without preoperative 5-ALA administration in an anaplastic astrocytoma CNS WHO 3. (B) A different example of autofluorescence in glioblastoma in the yellow to orange wavelength range. (C) Intermediate autofluorescence in an Oligodendroglioma CNS WHO 3. Large image scale bars = 450 µm; count scale: 0–255 counts. [file Image_5.tiff]
